# Advancing the WEFE nexus: Expert insights on implementation and challenges

**DOI:** 10.1371/journal.pone.0350133

**Published:** 2026-07-06

**Authors:** Nikolaos Mellios, Maria Vrachioli, Georgia Tseva, Bettina Renner, Chrysaida-Aliki Papadopoulou, Giannis Adamos, Chrysi Laspidou

**Affiliations:** 1 Civil Engineering Department, University of Thessaly, Pedion Areos, Volos, Greece; 2 Chair Group of Production and Resource Economics, Technical University of Munich, Alte Akademi, Freising, Germany; 3 Geography Department, Harokopio University, Eleftheriou Venizelou Ave., Athens, Greece; 4 School of Rural, Surveying and Geoinformatics Engineering, National Technical University of Athens, Zografou GR, Athens, Greece; 5 Civil Engineering Department, Aristotle University of Thessaloniki, Thessaloniki GR, Greece; Queensland University of Technology, AUSTRALIA

## Abstract

This paper builds on the Water-Energy-Food-Ecosystems (WEFE) nexus approach through insights gleaned from interviews with project coordinators involved in nexus-oriented research and initiatives. By employing a structured interview format, empirical evidence highlighting the perspectives of experts on the methodologies, practices, and challenges within the nexus framework was gathered. The findings indicate that, despite growing understanding of the complexities of the WEFE nexus, its translation into practice remains limited across many project contexts. Interview participants emphasized the importance of recognizing the interdependencies among water, energy, food, and ecosystems to enhance resource efficiency and resilience against climate change. Key opportunities identified include the development of innovative technologies and nexus-specific indicators aimed at improving nexus understanding, fostering stakeholder engagement, and supporting collaborative decision-making. The study also identified pressing challenges, such as data availability, methodological alignment, and the integration of nexus activities with broader policy frameworks. Overall, the analysis examines how the WEFE nexus is conceptualized and operationalized in practice, and how analytical and governance-related challenges shape its implementation in European research and innovation projects. The insights gained from this research underscore the need for enhanced governance, effective communication, and strategic collaboration among stakeholders to capitalize on the full potential of the WEFE nexus. This paper contributes to the discourse on integrated resource management by providing a real-world perspective on the application, efficacy, and impact of the WEFE nexus framework, thereby enriching the existing knowledge stock on sustainable resource utilization.

## 1. Introduction

Water, energy, food, and ecosystems are intricately interconnected components that form the basis of sustainable development and efficient resource management. The WEFE nexus approach has emerged as a key framework to address complex resource and development challenges for over one decade [1–5]. It aims to reduce trade-offs and enhance the efficiency of the entire system through synergies, while maintaining the integrity of our ecosystems [6]. In recent years, significant progress has been made in developing methodologies to model the WEFE nexus, aiming to enhance understanding on these complex interactions and support integrated decision-making. State-of-the-art methodologies in WEFE nexus modeling encompass a diverse array of analytical tools and approaches [7–10]. Recent advancements in modeling the WEFE nexus have led to the development of methodologies that utilize advanced computational models, remote sensing technologies, data analytics, and interdisciplinary frameworks to capture the complex interconnections among these resources. For instance, data-driven modeling approaches have been employed to facilitate understanding of the WEFE nexus’s physical and social dynamics [11]. Additionally, interdisciplinary science efforts provide new understanding of the interdependence of food, energy, and water sectors, supporting coordinated management for improved sustainability [12]. Dynamic modeling techniques, such as system dynamics and integrated assessment models, enable researchers to simulate various scenarios and assess the potential impacts of policy interventions across WEFE sectors [13–15].

At its core, the concept of interdisciplinarity is straightforward, and it arises from the belief that effective responses to significant environmental challenges require the amalgamation of knowledge and expertise across various fields. Without such integration, there’s a significant risk of failing to mitigate threats and inconsistencies; thus, missing important opportunities for integrated and efficient resource use. This essentially suggests that traditional, sector-focused methods are inadequate for tackling the complex and interconnected issues related to natural resource management, as highlighted by Scoones & Stirling [16]. For this reason, the nexus approach for sustainable development and management of natural resources has gained ground in both scientific and policy-making circles during the last years. However, it still faces a limited uptake in practice despite its promising potential when applied at a research level. Bridging the gap between nexus frameworks, policy recommendations, and decision-making is still a procedure that constantly matures towards the establishment of well-informed nexus-oriented policy schemes.

The landscape of nexus research and innovation has been significantly influenced by the emergence of enhanced funding projects and open calls, which have catalyzed advancements in monitoring and modeling techniques within the domain. Notable literature contributions have highlighted the growing momentum of the nexus approach, emphasizing its role in supporting stakeholder engagement, amplifying policy coherence and fostering systems integration [17; 18]. Scholarly discussions highlight the intricate interplay between the nexus typology and the assessment of societal, technological, and political dynamics across sectors [19]. Challenges related to monitoring and modeling techniques within the nexus framework have been acknowledged, particularly as tools of high complexity, making it increasingly difficult to capture the full spectrum of interlinkages among diverse system components [20]. Efforts to enhance integrated planning within the nexus approach have been advocated as a pivotal strategy for addressing complexities and challenges inherent in understanding and operationalizing nexus concepts. Key considerations such as spatial scale [21], data-driven decision-making [22], dynamic stakeholder engagement [23], differentiated political and cultural backgrounds, play essential roles in advancing the effective implementation of the nexus typology.

The aim of this article is to present insights and knowledge directly extracted from experts actively engaged in nexus-oriented research projects and initiatives, via interview campaigns conducted with project coordinators that are focused on the nexus approach. Throughout this paper, the Water–Energy–Food–Ecosystems (WEFE) nexus is adopted as the primary analytical framework; the term “WEF” is used only when explicitly referring to tools or studies that employ a WEF framing. The rationale behind the interview approach relies on the fact that it: i) fosters a direct interaction with the interviewees and allows for gaining rich and detailed data; ii) enables interviewers to establish rapport and trust with the participants, which can enhance the quality and depth of the acquired information; iii) offers the fertile ground to gather sensitive or hard to obtain information. Unlike review papers which compile and synthesize existing findings from various sources, this paper offers firsthand, empirical evidence and perspectives that add a unique and valuable dimension to the understanding of the nexus framework. It seeks to delve into the practical experiences, challenges, and strategies identified by those at the forefront of implementing and managing projects that integrate multiple sectors and disciplines to address complex environmental and resource management issues. By grounding its analysis in direct conversations with scientists and project coordinators, the paper aims to contribute fresh, real-world insights to the academic and practical discourse on sustainable resource use, thereby enriching the existing knowledge stock with better understandings of the application, efficacy, and impact of the nexus approach.

The interview questions developed were significantly influenced by the methodological framework presented in Albrecht et al. [8], which systematically reviewed approaches for assessing the Water-Energy-Food nexus. Albrecht et al. [8] highlighted the limitations of existing nexus methodologies, particularly their tendency to remain confined to disciplinary silos and their lack of integration of social and political dimensions. While Albrecht et al. [8] review methods developed within a WEF nexus framework, the present study builds on these insights by adopting a broader Water–Energy–Food–Ecosystems (WEFE) perspective. Taking these findings into account, structured interview questions in this study were designed to explore both technical and governance aspects of nexus-oriented projects, including the models used, stakeholder engagement strategies, and challenges faced in implementing an integrated nexus approach. By aligning the interviews with the analytical gaps, i.e., data and research requirements, normative attributes, identified in Albrecht et al. [8], this study aims to capture a more comprehensive perspective on the nexus, addressing both methodological robustness and practical implementation challenges. While existing reviews, such as Albrecht et al. [8], focus on classifying and evaluating WEF nexus methods, the present study complements this literature by examining how such methods are actually selected, adapted, and applied in real-world WEFE projects. In this sense, the contribution of this study lies not in proposing new analytical tools, but in providing empirical insight into the practical use and perceived limitations of existing nexus methods from the perspective of project practitioners.

The structured interview methodology is based on the formulation of specific questions and the elicitation of specific responses by the interviewee [24; 25] while the sequence and wording of questions as well as the duration of the interview can be organized a priori [26]. A structured interview includes a predefined number of standardized questions [27; 28] that can be replied by selecting one or more possible responses from a fixed range of answers (e.g., multiple-choice questions). It thus facilitates the acquisition of quick and explicit answers that can be further elaborated and statistically analysed by researchers; something that allows for comparisons to be conducted and sound conclusions to be formulated.

Building on this methodological approach, this study empirically examines how the Water–Energy–Food–Ecosystems (WEFE) nexus is conceptualized and operationalized in practice within European research and innovation projects. Specifically, the study addresses the following research questions:

**RQ1**: How do project coordinators conceptualize the WEFE nexus and its core components in practice?;

**RQ2**: Which analytical approaches, models, and data sources are used to operationalize the WEFE nexus within projects?;

**RQ3**: What challenges and opportunities do practitioners identify when implementing the WEFE nexus approach?;

**RQ4**: How do these challenges differ between analytical (data and modelling) dimensions and governance- and policy-related dimensions?

To address these research questions, the manuscript is structured as follows. Following the introduction, the methodological approach is described, detailing the design and analysis of structured interviews used to capture practitioners’ perspectives. The results section presents the empirical findings, combining quantitative synthesis of interview responses with qualitative thematic analysis to explore patterns, challenges, and enabling factors related to WEFE implementation. These findings are then discussed in relation to existing WEFE nexus literature, highlighting implications for research and practice, and the manuscript concludes by summarizing the main contributions and outlining directions for future work.

## 2. Materials and methods

### 2.1. *Selection of nexus-related projects from the NexusNet project database*

NexusNet is an international consortium of researchers including academic and research institutes, policy makers, and the business community to deepen the understanding of how the nexus promotes policy alignment and biophysical synergies across water, energy, food, ecosystems, climate, health, land, etc., sectors. The project employs interdisciplinary and transdisciplinary methodologies to evaluate nexus-aligned practices, incorporating input from key stakeholders and maintaining engagement with ongoing and completed nexus-focused projects. Moreover, it underscores that integrated modeling, transdisciplinary decision-making, and comprehensive nexus analysis—which incorporates both biophysical and socio-economic factors—are essential for thoroughly investigating current challenges and identifying viable solutions. The project is organized into six Working Groups to focus on different aspects of the nexus approach, with each group concentrating on specific topics and objectives to further the overall goals of the network. The current analysis is an outcome of WG1 which focuses on the theme titled “Monitoring and modeling the nexus” and especially of the subtask that has at its core the assessment of monitoring and modeling nexus techniques to suggest improvements and state-of-the-art approaches and to develop a nexus typology.

After selecting projects from the NexusNet project database, an invitation email was sent to 50 nexus project coordinators, together with a brief description of the interview aim and a consent form. From this pool of project coordinators, 15 replied positively and expressed their willingness to participate in the interviews. This study involved voluntary interviews with adult project coordinators focusing on professional practices and project experiences within the WEFE nexus framework. According to NexusNet guidelines, this type of research did not require formal ethics committee approval. Nevertheless, written informed consent was obtained from all participants prior to the interviews.

In accordance with the principles outlined in the consent form, which prioritize the anonymity of project names and project coordinators, an overview of the projects is presented in Table 1.

**Table 1:** Overview of the nexus projects whose coordinators engaged in the interviews.

Project	Description
Project 01	Engages with a consortium of experts to explore socio-economic potential of long-term Water-Energy-Food-Ecosystem (WEFE) nexus solutions to foster innovation and sustainable development
Project 02	Focuses on the energy sector, investigating the transition towards a low-carbon economy and its interactions with water and land resources
Project 03	Employs the approach of societal metabolism to analyze fund-flow relationships within the WEFE nexus, seeking to understand resource allocation and utilization
Project 04	Delves into agricultural reuse of treated water, connecting ecosystem and climate elements with water quality and quantity
Project 05	Examines nexus grand challenges in four Mediterranean countries through nexus ecosystem labs to facilitate local stakeholder collaboration
Project 06	Addresses WEFE components with a focus on climate, land, and soil dynamics, recognizing their interplay and impacts on the nexus
Project 07	Aims to catalyze a shift from conceptual thinking to practical resilient actions in the WEFE nexus using visual tools
Project 08	Places emphasis on ecosystem services in supporting the WEFE nexus and how the nexus influences ecosystems
Project 09	Targets circular water solutions, addressing water-energy interactions and assessing technology impacts on ecosystem health
Project 10	Deals with a waste-to-fuel initiative within the Food-Energy-Water nexus, employing living labs for enhanced understanding and collaboration
Project 11	Aspires to manage resilient nexus systems through participatory systems dynamics modeling
Project 12	Focuses on resource valorization within the water cycle to generate economic value, considering impacts on ecosystem health and climate change
Project 13	Supports fostering WEFE nexus connections in Mediterranean’s northern African countries, optimizing innovation systems for sustainable resource utilization
Project 14	Dedicated to energy recovery from waste streams, with a primary emphasis on the water-energy nexus
Project 15	Contributes to a multifaceted approach encompassing economic, social, hydrological, and ecological dimensions, focusing on resilient and productive agro-ecosystems

### 2.2. *Interviews analysis approach*

The interviews were executed online through Microsoft Teams between April 28 and May 11, 2023. Each interview, conducted in English, lasted approximately 30 minutes. The sessions were held during regular working hours, with all participants volunteering without any financial compensation. Interview recordings were stored securely, adhering to all relevant privacy regulations to ensure confidentiality and protection of data.

A structured interview format was employed for the online interviews, comprising standardized open-ended questions and predetermined multiple-choice questions designed to gather specific, relevant information pertaining to the nexus approach. This method ensured consistency and comparability across all participants’ responses, facilitating a robust quantitative and qualitative analysis. During the interviews, the interviewers actively engaged with participants by asking follow-up questions based on their initial responses. This technique promoted a more in-depth discussion, enabling the collection of richer qualitative data.

The interview protocol was informed by gaps identified in existing methodological reviews of the nexus literature, particularly Albrecht et al. [8], which highlight challenges related to data availability, model integration, and policy relevance. While Albrecht et al. focus on evaluating nexus methods based on their theoretical characteristics, the present study adopts a complementary perspective by examining how these methods are actually used and perceived by practitioners in real WEFE projects. The interview protocol covered nine domains: (i) nexus components addressed; (ii) motivations for adopting a nexus approach; (iii) analytical approaches; (iv) models applied; (v) data types and sources; (vi) stakeholder engagement; (vii) indicators used or developed; (viii) uncertainty or robustness analysis; and (ix) implementation challenges. The compiled list of interview questions is provided in S1 Appendix.

Quantitative analysis of structured interview responses was used to identify cross-project patterns related to how the WEFE nexus is conceptualized and operationalized, including nexus components addressed, analytical approaches, models, and data sources (RQ1–RQ2). Qualitative thematic analysis was used to explore practitioners’ experiences, perceptions, and reflections regarding implementation challenges and opportunities (RQ3–RQ4).

Upon completion of the interviews, all recordings were meticulously transcribed, creating a verbatim account of each session. A researcher supporting the Working Group 1 leader and co-leader, handled the transcription process. The transcriptions were then summarized to highlight key points and issues. This step was crucial for identifying patterns and commonalities across the datasets, essential for subsequent quantitative analysis.

#### 2.2.1. Quantitative analysis.

The quantitative analysis hinged primarily on frequency analysis and visualization outputs. Frequency analysis was conducted to determine the prevalence of specific responses and themes within the dataset. By calculating the frequency of responses to each question, we were able to identify common practices, methodologies, and key areas of focus among the project coordinators. This analysis provided a statistical overview of trends and patterns that emerged from the interviews. To visually represent these findings, a Sankey diagram and bar charts were utilized. The Sankey diagram was particularly effective in illustrating the flow and interconnections between different components of the nexus approach and was designed by using the SankeyMATIC web-based tool ([href:https://sankeymatic.com/]https://sankeymatic.com/). It helped highlight significant synergies and trade-offs, providing a clear and intuitive representation of complex relationships and resource flows. Bar charts were employed to depict the distribution of responses related to various nexus methodologies, models used, stakeholder engagement levels, and data types. These charts facilitated the identification of dominant practices and comparative trends among the different respondents.

#### 2.2.2. Qualitative analysis.

A ‘mot à mot’ (word-for-word) transcription of the material was performed using the Transkriptor software ([href:https://app.transkriptor.com/signin]https://app.transkriptor.com/). Based on the material transcription the interviewees’ responses were organized into a spreadsheet to enable coding and thematic analysis of the material. Furthermore, as the interview campaign objectives were reflected on several topics related to the sociotechnical environment the projects are implemented, a new research question was formulated as the basis for coding and thematic analysis: “What are the key elements that the projects prioritize to reach their aims?”.

Focused on the research question formulated, as well as on the interviewees’ responses, a thematic analysis was performed to uncover important patterns in the interviewees’ responses. While interviewees’ responses indicated clearly the selection of distinct strategies in the implementation of the projects they represented, they also linked these with specific positive or negative attributes. Thematic analysis was chosen as the most suitable way to analyze responses in open-ended questions based on the interviewee’s experiences and not interpretations, as these were not explored through the interviews. In this context, inductive coding was implemented, in which codes were created based on the qualitative data itself [29]. Derived from the coding process, the following themes and subthemes were developed for the analysis of the data (Table 2). The analysis took into consideration also the sentiment dimension as quotes by the interview –hence, whether a theme was addressed as an opportunity or a challenge.

**Table 2:** Dimensions, themes and subthemes developed under the qualitative analysis framework.

Dimensions	Themes	Subthemes
Opportunities	New knowledge	Innovative technology
		New data
		New assessment methodologies
		New Business Models
		Nexus specific indicators
	Ecosystem Building	New synergies
		Stakeholders’ Involvement
Challenges	Data availability and Sharing	Data availability
		Data accessibility
		Data sharing
		Data reliability
	Modelling methodologies Alignment	Linking models to sectoral data difficulties
		Linking models difficulties
		Nexus modelling difficulties
		Resources Use conflict
	Integration with development policies	Communication gaps
		Knowledge transfer
		Direct Impact
		Sustainable Funding
	Externalities	Political Instability
		Development Stage

To analyse the data, the survey responses were integrated into NVIVO software ([href:https://lumivero.com/products/nvivo/]https://lumivero.com/products/nvivo/) for comprehensive examination. After careful examination of the material, the open questions’ responses were introduced as open questions to the software (see S1 Appendix). For the qualitative analysis, Question 1 concerning Nexus Components and Question 8 pertaining to the conduction of uncertainty or robustness analysis were formulated as closed questions to facilitate the structured analysis necessary for further relational exploration of the data. In contrast, although originally designed as structured questions, Questions 3.1–3.5 regarding the methodologies employed within the projects and Questions 5.1–5.2 related to the types and sources of data were presented as open-ended questions. This methodological choice aimed to capture qualitatively the emergent themes articulated in the interviewees’ justifications. Upon detailed examination, responses were subsequently categorized according to predefined thematic frameworks.

## 3. Results and discussion

### 3.1. *Quantitative analysis results*

#### 3.1.1. Nexus components, synergies, trade-offs and aims for applying the nexus approach.

The first survey question (Q1) aimed to identify the key nexus components of the respondents’ projects. The analysis revealed that water, energy, food, climate, and ecosystems are the predominant components addressed across the majority of projects studied. Ecosystems are frequently recognized as an overarching factor critical for evaluating the environmental impacts of new nexus solutions. Furthermore, other aspects such as land and soil, waste management, and health, although not always direct focal points, are considered in various capacities within the projects. Climate emerges as a significant nexus driver, notably highlighted by two interviewees who emphasized its influential role in shaping nexus interactions. Additionally, there is a geographical focus evident in some projects aiming to enhance WEFE nexus synergies specifically among Mediterranean countries. Several projects also engage in exploring the interconnections between water, waste, and energy, underlining the complexity and multifaceted nature of nexus components.

The second question (Q1.1) explored the identification of nexus hotspots, particularly focusing on critical synergies and trade-offs. Interviewees highlighted numerous examples, such as the interplay between food production and renewable energy generation, harnessing energy from wastewater streams, and repurposing treated wastewater for agricultural irrigation. Moreover, participants pinpointed significant areas that extend beyond direct nexus connections. One such area encompasses the requirement for societal change management to accommodate fresh perspectives on sustainable resource management. Another encompasses the necessity for aligning business models and establishing regulatory frameworks and governance, especially when considering the utilization of materials recovered from waste streams.

Inquiry Q2 was geared towards exploring the foundational motivations that drive the incorporation of a nexus approach within the projects. The findings reveal that a prevailing objective among projects is the enhancement of resource efficiency, bolstering sustainability, and fostering adaptability and resilience in response to climate change. Within the “Other” category, respondents emphasized priorities like “climate change mitigation,” “food/resource security,” “impact”, and “circular economy”. Notably, the pursuit of resource efficiency encompasses not only Water-Energy-Food-Ecosystems (WEFE) resources but extends to other dimensions such as land and minerals. When considering the actions geared towards achieving resource efficiency, prominently spotlighted actions include the reutilization of wastewater for agricultural irrigation, as well as the recovery of nutrients and energy from wastewater and waste streams. Additionally, it’s noteworthy that several objectives are interconnected; for instance, the pursuit of enhanced policy integration necessitates a foundational comprehension of sustainability principles, demonstrating the interdependent nature of these aspirations.

**Fig 1:**
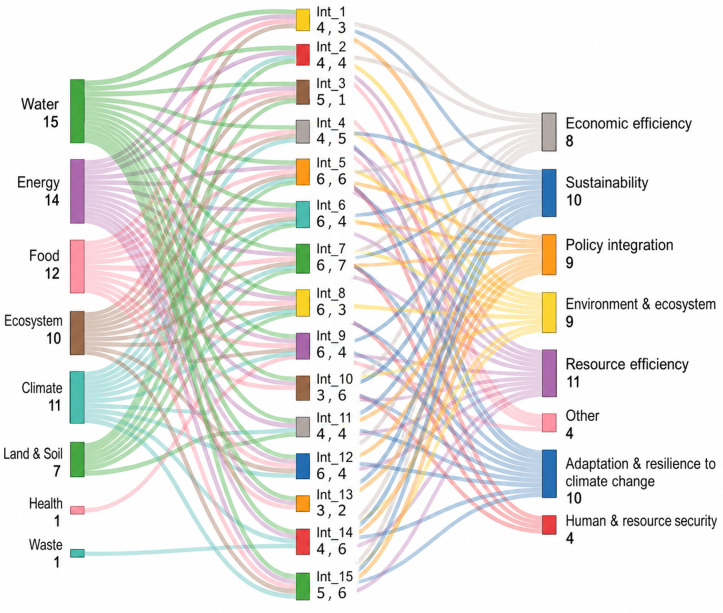
A Sankey diagram illustrating the 15 interviewees’ responses with regards to: i) what are the nexus components their projects deal with (left part) and ii) the aims of using the nexus approach (right part). In the central part of the diagram, the responses of the interviewees are depicted, illustrating how they are distributed among the nexus components (left number) and the aims for using the nexus approach (right number).

Fig 1 illustrates the interviewees’ responses on the nexus components their projects include and the aims of using the nexus approach.

#### 3.1.2. Methodologies to model the nexus.

Methodological approaches for evaluating and modeling the nexus draw from a wide range of disciplines, each offering distinct tools to address the complexity of interlinked systems. Systems analysis includes techniques such as system dynamics and causal loop diagrams, which help in understanding feedbacks and behavior over time. Integrated modelling brings together sector-specific models into a cohesive framework, enabling scenario analysis and policy assessment across multiple domains. Environmental management approaches, such as life cycle assessment and footprinting, evaluate the environmental impacts of processes and interventions across their full life span. Economic methods, including cost-benefit analysis and value chain analysis, provide insights into trade-offs, resource allocation, and economic viability. Statistical techniques, such as regression analysis and data mining, are used to detect patterns, correlations, and trends within large and often heterogeneous datasets. In the survey, participants were asked to indicate which of these methodological families they applied within their projects, often drawing on multiple approaches to capture the multifaceted nature of the WEFE nexus.

**Fig 2:**
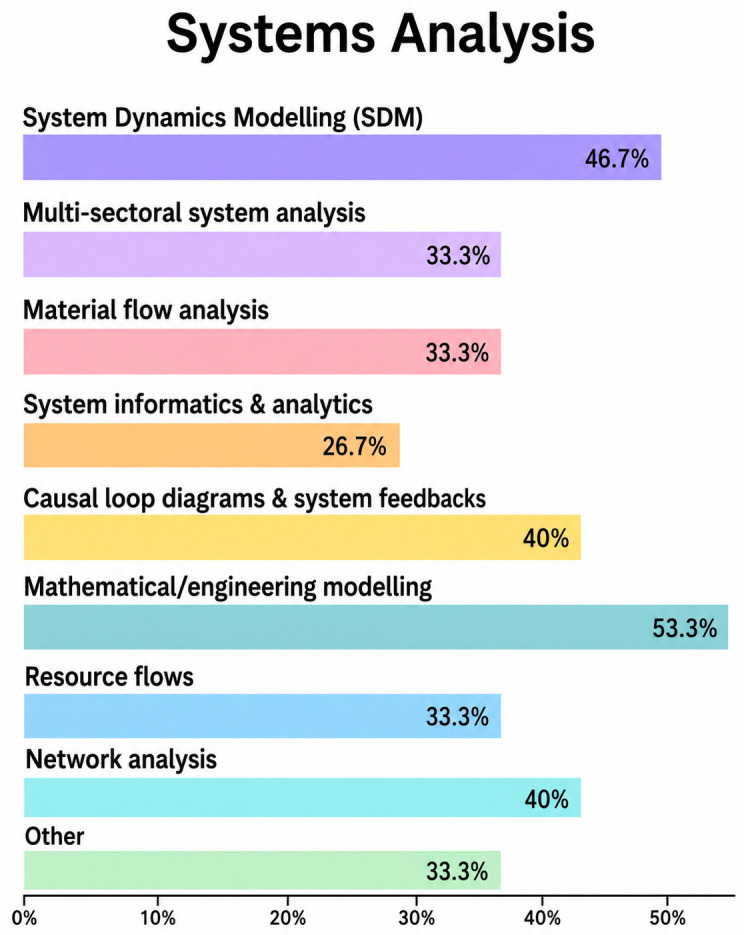
Bar chart illustrating the frequency of responses from the interviewees in Systems Analysis.

*3.1.2.1 Systems Analysis:* The insights gathered from respondents concerning Systems Analysis methodologies (Q3.1) offer a comprehensive view of the diverse approaches employed within this field (Fig 2). Mathematical and engineering modelling emerged as a key technique, reflecting the importance of quantitative tools in capturing and predicting dynamic system behaviors with a high degree of precision. System dynamics modelling was also widely cited, highlighting its value in simulating feedback loops and long-term interactions across system components.

Several participants noted the utility of causal loop diagrams and network analysis, both of which support the visualization of interconnections and the identification of emergent system properties. Other approaches, such as resource flow mapping, material flow analysis, and multi-sectoral system analysis, were recognized for their ability to track resource allocation and interactions across sectors, thus supporting a more integrated perspective on system functioning.

The use of system informatics and analytics was also discussed, particularly in the context of leveraging digital tools and information systems to manage and interpret complex datasets. Beyond these, interviewees described a range of innovative and interdisciplinary methods—including agent-based modelling, the design and analysis of nature-based solutions, and applications of artificial intelligence such as machine learning and semantic platforms. These varied responses reflect growing tendency toward hybrid modelling strategies and highlight the continuous evolution of systems analysis within the nexus field.

**Fig 3:**
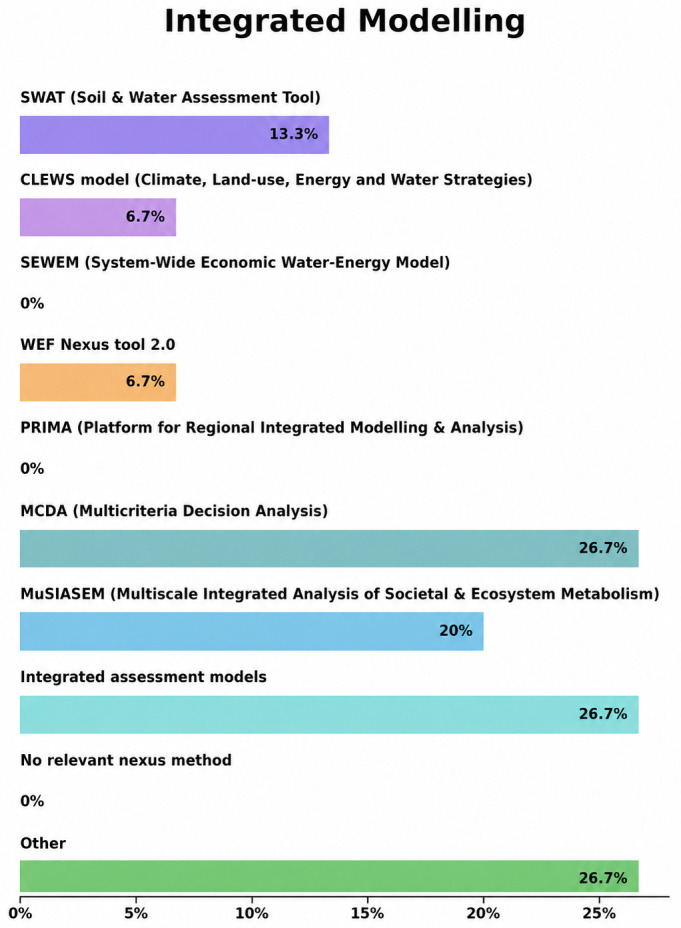
Bar chart illustrating the frequency of responses from the interviewees in Integrated modelling.

*3.1.2.2 Integrated Modelling:* The responses concerning the methodologies employed within Integrated Modelling (Q3.2) shed light on the diverse landscape of approaches utilized by different projects (Fig 3). Many project coordinators emphasized the value of Multicriteria Decision Analysis (MCDA) [30] as a practical tool for navigating complex decision-making environments. By allowing various criteria—social, economic, and environmental—to be evaluated simultaneously, MCDA supports more balanced and inclusive planning processes. Similarly, the use of Integrated Assessment Models reflected a clear preference for holistic frameworks that combine insights from multiple disciplines to address the interconnected nature of water, energy, food, and ecosystems.

Interestingly, a significant number of responses pointed to tools falling under the “Other” category, revealing a strong appetite for methodological innovation. These included models such as WATNEEDS [31], FREEWAT [32], and WEAP [33], which are often tailored to the unique needs of specific projects or regions. This suggests that rather than relying solely on standardized models, project teams are frequently customizing or combining tools to better capture the nuances of the systems they work within.

The use of MuSIASEM [34]—an approach focused on analyzing the metabolic patterns of societal and ecosystem interactions—demonstrated a growing interest in understanding the broader human-environment interface. Meanwhile, models like SWAT [35] were highlighted for their capability to evaluate interactions between soil and water, pointing to the importance of hydrological processes in many nexus studies.

Although used less frequently, specialized tools such as CLEWS [36] and the WEF Nexus Tool 2.0 [19] were acknowledged for their ability to map out interdependencies across climate, land use, energy, and water, emphasizing the added value of models designed specifically for nexus applications. On the other hand, tools like SEWEM [37] and PRIMA [38] did not appear in participants’ responses, suggesting either limited awareness or applicability in current projects—an observation that could point to areas for further capacity building or methodological adaptation.

Overall, the findings illustrate a landscape in which project teams are actively engaging with both well-established and emerging modelling techniques, striving to develop integrated tools that can more effectively support the complex demands of nexus planning and policy.

**Fig 4:**
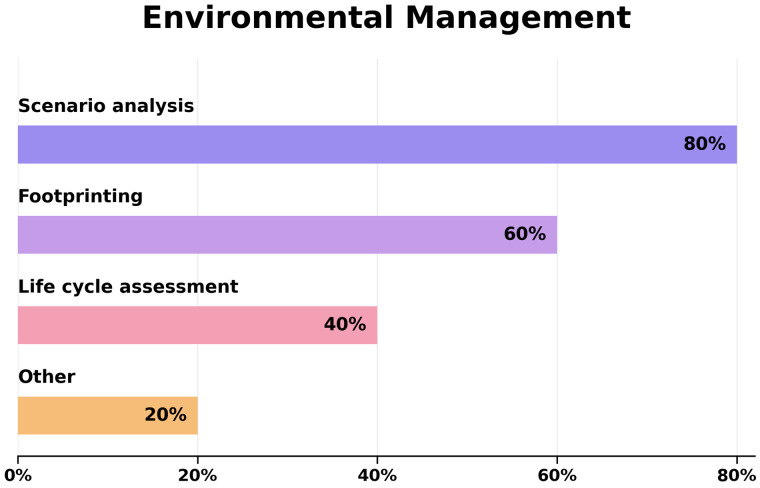
Bar chart illustrating the frequency of responses from the interviewees in Environmental Management.

*3.1.2.3 Environmental Management:* In exploring the methodologies applied within the thematic area of environmental management, interviewees provided crucial information of the tools shaping nexus-related decision-making (Fig 4). Scenario analysis stood out as the most widely adopted approach, reflecting its central role in navigating environmental complexity. By enabling the exploration of multiple future pathways, this method allows project teams to anticipate outcomes under varying conditions and inform more resilient planning strategies.

Footprinting was also commonly employed, underscoring the importance placed on quantifying environmental impacts—whether related to water use, carbon emissions, or broader ecological footprints. This approach helps teams measure the sustainability of their interventions and track progress toward environmental goals.

Life Cycle Assessment (LCA) was another key methodology, recognized for its capacity to evaluate environmental impacts across the entire life span of products or processes. From resource extraction through production and disposal, LCA provides a holistic view that is essential for understanding long-term environmental trade-offs and benefits.

In addition to these core methods, some participants described the use of more specialized tools, grouped under the “Other” category. These included uncertainty analysis using stochastic sampling techniques, political and governance analysis to examine institutional contexts, and human and environmental risk assessments. Collectively, these tools point to an evolving methodological landscape—one that increasingly integrates scientific analysis with socio-political awareness to address the multifaceted challenges of environmental management within the nexus framework.

**Fig 5:**
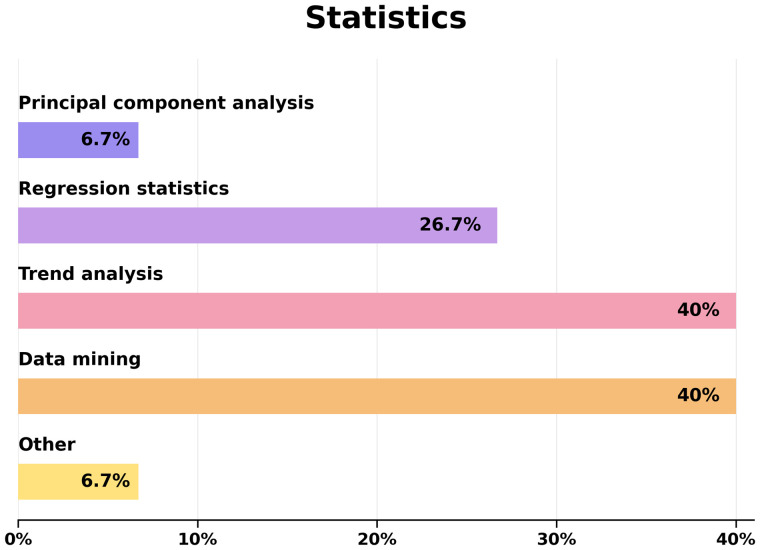
Bar chart illustrating the frequency of responses from the interviewees in Statistics.

*3.1.2.4 Statistics:* The responses to Question 3.4 reveal the diverse range of statistical methodologies employed across projects, offering valuable insight into how analytical tools are used to interpret complex nexus data and support informed decision-making (Fig 5). Trend analysis and data mining emerged as particularly valuable tools—used not only to trace how variables evolve over time, but also to extract hidden insights from large, multifaceted datasets. These techniques demonstrate a strong commitment to harnessing data for evidence-based planning and to making sense of intricate resource interdependencies.

Regression analysis was also commonly applied, with participants highlighting its utility in exploring the relationships between variables. Whether examining how water use affects food production or how energy demands shift under different climate scenarios, regression models offer a way to quantify and predict these connections, enhancing both understanding and forecasting capacity.

In a few cases, projects employed principal component analysis (PCA) to distill complex datasets into their most informative elements. By reducing dimensionality while preserving critical variance, PCA enables more streamlined and interpretable analyses—particularly useful when working with large, multivariate datasets.

Beyond conventional methods, some interviewees shared their use of advanced techniques falling under the “Other” category. These included the incorporation of agent-based modelling components into artificial intelligence and machine learning workflows, shaping a forward-looking approach that blends traditional statistics with emergent computational tools. Collectively, these methodological choices reflect the evolving analytical sophistication of nexus research, as teams increasingly seek to leverage the full power of data in navigating cross-sectoral challenges.

**Fig 6:**
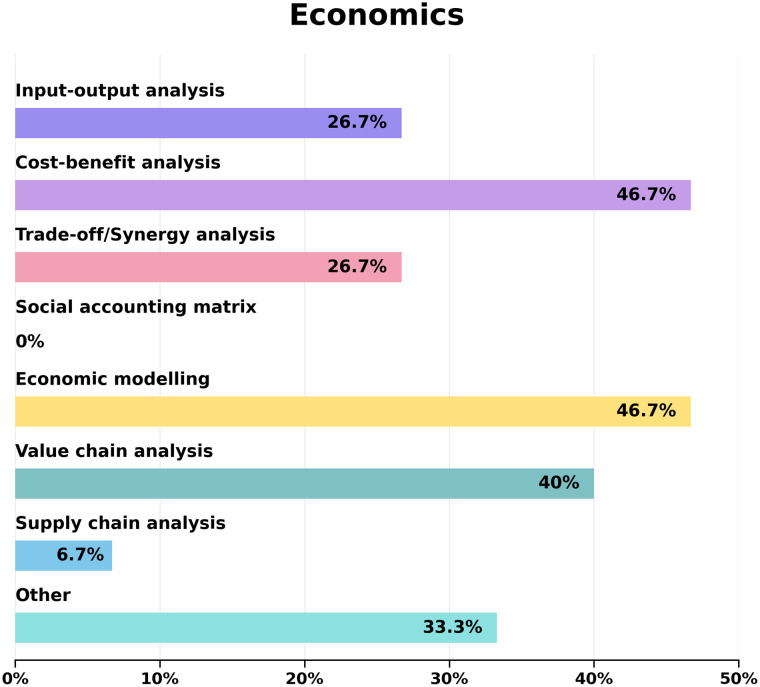
Bar chart illustrating the frequency of responses from the interviewees in Economics.

*3.1.2.5 Economics:* The outcomes from the methodologies utilized within the area of Economics (Q3.5) provide an insightful perspective into the analytical tools adopted by diverse projects (Fig 6). Cost-benefit analysis and economic modelling emerged as the most widely applied techniques, each serving distinct yet complementary purposes. While cost-benefit analysis helps weigh the advantages and disadvantages of proposed actions in economic terms, economic modelling offers a broader framework for simulating behaviors and projecting outcomes under various policy or market scenarios.

Value chain analysis was also frequently cited, reflecting its utility in mapping the flow of value through production systems and identifying opportunities for efficiency or sustainability improvements within specific sectors. Many projects extended their toolkit even further, drawing on a diverse set of methods grouped under the “Other” category. These included technoeconomic modelling, macroeconomic analysis, input-output profiling, and business models tailored to nature-based solutions. Such diversity highlights a strong inclination among participants to experiment with innovative economic frameworks that align with the complexities of nexus planning.

Input-output analysis and trade-off/synergy analysis were also noted for their roles in examining sectoral interdependencies and exploring how decisions in one area may generate benefits—or impose costs—on others. Meanwhile, supply chain analysis, though mentioned less frequently, was acknowledged for its relevance in understanding the logistical and economic processes that underpin resource flows.

Interestingly, the social accounting matrix was not referenced by any participants, which may suggest limited familiarity with this method or a perception of its lower relevance to current nexus initiatives. Overall, the findings underscore a growing sophistication in economic thinking, with project teams increasingly turning to both established and emerging tools to navigate the intricate interplay between environmental goals and economic outcomes.

#### 3.1.3. Models used for analyzing nexus components.

The interviewees provided detailed insights into the models used across the nexus components in their projects. Below is a summary explaining the responses, indicating which interviewee provided each piece of information.

Interviewee I01 identified the WEF Nexus 2.0 model [19] as the most widely used tool across the assessed projects. This model is central for addressing the interdependencies within the water-energy-food nexus. Interviewee I02 mentioned the use of ENERGYPLAN [39] and EURO-CALLIOPE [40], tools specifically designed for modeling and analyzing energy systems within a broader nexus framework. Interviewee I03 highlighted the Multi-Scale Integrated Analysis model, which focuses on analyzing complex systems at multiple scales, demonstrating its utility in nexus modeling. Interviewee I04 referenced a new modeling tool developed in the form of a platform. This tool is used to simulate the behavior of various solutions under different environmental conditions, indicating a novel approach within the project. Interviewee I05 mentioned the models WATNEEDS [31] and FREEWAT [32], emphasizing their application in addressing specific environmental and resource management challenges. Interviewee I06 provided detailed information on ISI-MIP [41] and CMIP [42], two models that integrate biophysical data from sources such as LPJmL [43], PCR-GLOBWB [44], and WATERGAP2-2c [45], as well as socioeconomic data from GRDEM and DEMETRA. These models are valuable for assessing biophysical and socioeconomic dimensions of nexus components. Interviewee I07 outlined multiple models used for different components. For water, they listed SWAT [35], WEAP [33], HEC-RAS [46], and mGROWA [47], while food systems were modeled using water allocation models mentioned earlier. Ecosystems were primarily assessed using these models to evaluate the impact of Nature-Based Solutions (NBS). Integrated modeling was conducted using system dynamics modeling tools. Interviewee I08 noted that the INVEST model was used for water management, while ARIES (AI for Ecosystem Services and Sustainability) [48] was applied for crop and biomass production. Interviewee I09 discussed the adaptation of business models, such as the Eco Canvas, to integrate circular economy principles. This approach involved enhancing the business models to generate more circular and sustainable values. Interviewee I10 described the use of System Dynamics Modeling and Agent-Based Modeling, specifically in the context of addressing the Waste-Resource Paradox. These tools were utilized to explore the interactions between resources and waste in the nexus framework. Interviewee I11 provided a detailed account of water, energy, and climate modeling. For water, they mentioned the EDUMORIN model, developed by the University of Castilla-La Mancha, which uses remote sensing satellite data for accounting and footprinting. For energy, LCA (Life Cycle Assessment) was used for accounting and footprinting. Climate models involved the integration of multiple modeling tools (up to eight), and participatory system dynamics models were developed using their methodology called the Learning and Action Alliances Procedure. Interviewee I12 noted that no specific models were used for their project, indicating a lack of reliance on formal modeling tools. Interviewee I13 stated that the question could not be answered, as they were unable to provide information about the models used. Interviewee I14 focused on energy and heat recovery, describing the use of CFD (Competition of Free Dynamics) and the Lumped and Distributed Parameter Approach to model industrial systems. However, no information was available about the water treatment component of the project. (Note: I15 has been omitted as they do not contain explicit model-related information.)

This overview demonstrates a diverse range of tools and methodologies identified by the 15 interviewees, reflecting the complexity of the nexus components addressed in their projects. Each response highlights a different perspective or application, illustrating the multidisciplinary nature of the work.

#### 3.1.4. Data used towards quantifying the nexus.

**Fig 7:**
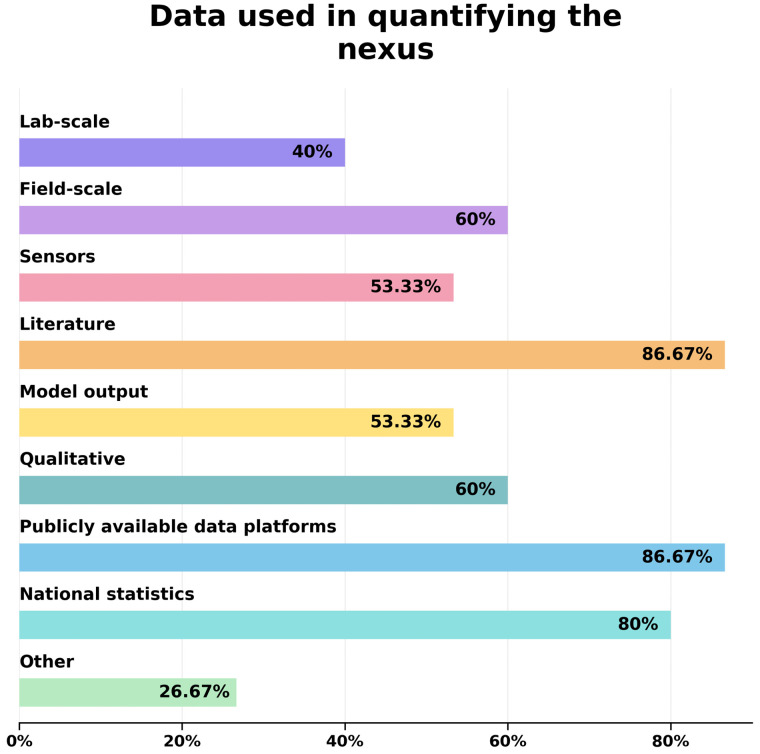
Bar chart illustrating the frequency of responses from the interviewees in Data used in quantifying the nexus.

**Fig 8:**
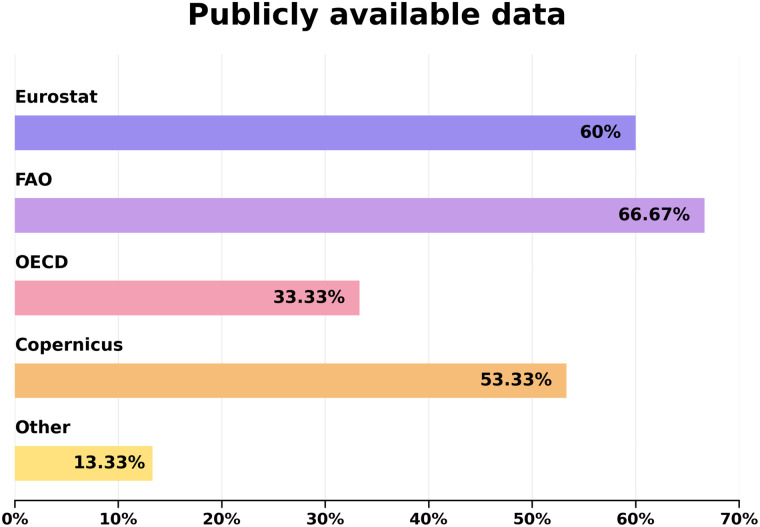
Bar chart illustrating the frequency of responses from the interviewees in publicly available data.

Data used in quantifying the nexus refers to the diverse range of information harnessed to measure and understand the intricate interactions within the nexus. This data encompasses a multitude of variables, including water availability, energy consumption, agricultural production, environmental impacts, economic factors, social dynamics, etc. Data quantification offers a comprehensive foundation for modeling, analysis, and informed decision-making. The integration of such data allows for a holistic view of nexus relationships, enabling the identification of synergies, trade-offs, and potential interventions to ensure sustainable resource management. In the following multiple-choice structured questions (Figs 7 & 8) the participants were asked to give information regarding the different types of data they use in their projects and from the publicly available data which platform they use, correspondingly.

The responses regarding the types of data used to quantify the nexus reveal a rich and varied landscape of sources, reflecting the multifaceted nature of nexus projects (Fig 7). Among the most common approaches, publicly available data platforms and literature were frequently highlighted, underscoring the value of drawing on open-access resources and existing research to inform and strengthen analytical work. These sources provide a strong foundation for nexus assessments, allowing projects to build upon established knowledge.

In addition to secondary sources, many projects rely heavily on firsthand data gathered through qualitative methods and field-scale observations. These include interviews, expert insights, and measurements taken directly from real-world environments—providing essential context and nuance that complement numerical data. Such information proves particularly valuable when exploring the socio-environmental dimensions of nexus systems.

Sensor-based monitoring and model outputs also play a key role in several projects. These tools offer dynamic, real-time insights into system behavior, enabling researchers to track variables like water flow, energy consumption, or ecosystem health with high precision. By simulating different scenarios or capturing live conditions, these methods help bring a deeper understanding of complex interactions to the forefront.

Controlled experiments conducted in lab-scale settings were also mentioned as an important data source, particularly when testing specific processes or technologies under replicable conditions. These controlled environments allow for fine-tuned exploration of cause-and-effect relationships within the nexus.

Lastly, some participants pointed to a variety of unconventional data sources. These included technical coefficients, pilot-scale studies, proprietary datasets collected during the project, and even data embedded within innovative business models like Ecocanva. This openness to diverse and sometimes experimental data streams reflects the innovative spirit of many nexus initiatives and their drive to uncover new ways of understanding and managing interlinked resources.

The outcomes of a multiple-choice question focusing on the utilization of publicly available data platforms provide valuable insights into the diverse sources that projects rely on for their information needs (Fig 8). The Food and Agriculture Organization (FAO) emerged as a widely used platform, reflecting a strong emphasis on agricultural and food-related data. Its global reach and credibility make it a go-to resource for projects focused on food systems and sustainable resource use.

Eurostat was also frequently mentioned, indicating the importance of official European Union statistics in providing consistent and comparable data across countries. Its role in enabling pan-European analysis is particularly valuable for projects working within or across EU member states.

Copernicus, the European Earth observation programme, featured prominently as well, illustrating the growing reliance on satellite-based environmental data. Through high-resolution imagery and geospatial datasets, Copernicus supports a deeper understanding of climate dynamics, land use, and natural resource changes—all central to nexus research.

The OECD was referenced by several participants as a source of international economic indicators, supporting broader assessments of policy and economic trends. Its inclusion shows how nexus projects often extend beyond environmental considerations to incorporate economic dimensions.

In addition to these main platforms, several other sources were noted under the “Other” category. These included national statistical offices like ISTAT, ICIMIT, and CMIT, as well as internal datasets collected within the projects themselves. Participants also mentioned specialized platforms such as the Joint Research Centre (JRC) and the European Soil Data Centre (ESDAC), alongside local meteorological services. This wide array of sources demonstrates the diverse data ecosystem that nexus projects draw upon, combining global, regional, and local perspectives to inform their work.

#### 3.1.5. Stakeholder engagement.

**Fig 9:**
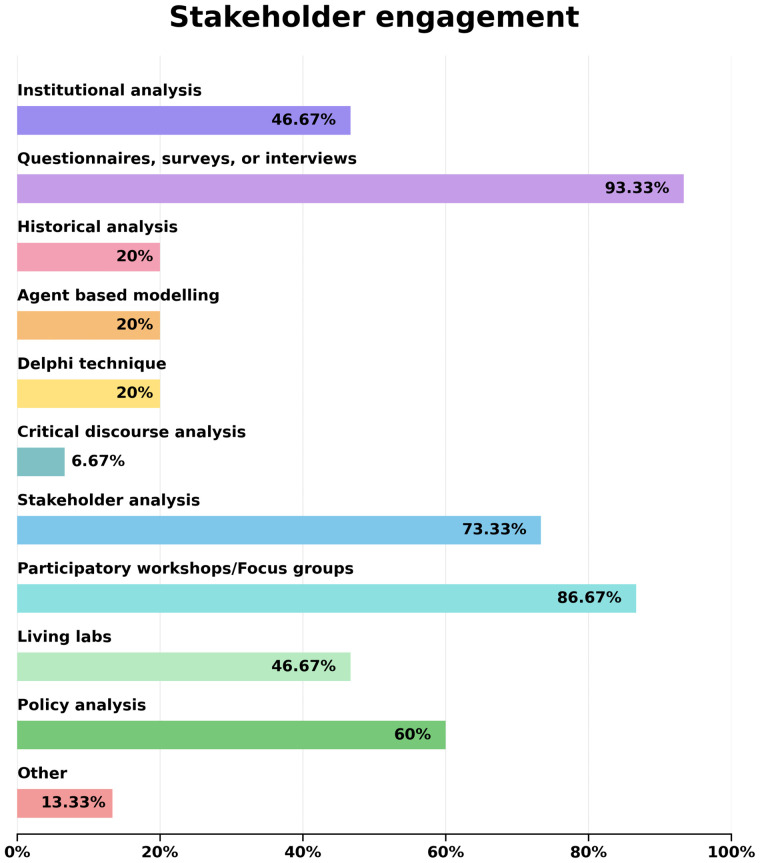
Bar chart illustrating the frequency of responses from the interviewees in Stakeholder engagement.

The insights from a multiple-choice question focusing on stakeholder engagement methods reveal the diverse strategies employed by projects to involve various stakeholders (Fig 9). The most widely used approaches were questionnaires, surveys, and interviews—tools that enable direct communication and allow researchers to gather targeted insights from diverse actors. These methods are particularly effective for capturing individual perspectives and informing evidence-based decision-making.

Participatory workshops and focus groups also featured prominently, underscoring the value of interactive spaces where stakeholders can exchange ideas, collaborate, and co-develop solutions. These forums not only foster mutual understanding but also help build trust among project participants, which is crucial for long-term engagement.

Stakeholder analysis was another frequently applied method, demonstrating a strategic effort to understand stakeholder interests, roles, and influence. This information allows project teams to design tailored engagement strategies that resonate with specific audiences.

Policy analysis was also commonly used, reflecting an awareness that stakeholder engagement does not occur in a vacuum but is deeply embedded within institutional and regulatory frameworks. By understanding the policy context, teams can better align their projects with existing governance structures and advocate for change where necessary.

Several projects employed living labs and institutional analysis, emphasizing the importance of testing solutions in real-world environments and examining how institutions facilitate—or hinder—collaboration. These approaches provide critical insights into the operational and structural dynamics that shape stakeholder relationships.

Less frequently, but still notably, projects utilized historical analysis, agent-based modelling, and the Delphi technique. These innovative methods offer unique ways of incorporating stakeholder input, whether by exploring past trajectories, simulating behavior, or seeking expert consensus.

A handful of respondents also mentioned alternative approaches under the “Other” category, including governance analysis, feedback sessions, result validation, and the Community of Practice model. These techniques highlight the diversity and creativity with which engagement is approached in different contexts.

Lastly, critical discourse analysis appeared occasionally, offering a more nuanced lens for examining how language, narratives, and power dynamics influence stakeholder interactions. Taken together, these varied methods illustrate a strong commitment to participatory, context-sensitive engagement as a cornerstone of effective nexus project design.

#### 3.1.6. Indicators developed or used in assessing the nexus.

When asked to identify any indicators developed or utilized in evaluating the nexus, it is worth noting that in 7 projects, no such indicators have been developed. However, several insightful approaches have emerged from different interviews. One approach suggested by Interviewee 03 (I03) involves creating a unified indicator by weighing a Multicriteria Analysis that takes diverse perspectives into consideration. Interviewee 05 (I05) reported ongoing efforts to develop their project’s indicator, which involves consolidating existing indicators and introducing new dimensions, including Ecosystem services. In the process of development is the WEFE nexus Footprint indicator, as shared by Interviewee 06 (I06). Project discussions have proposed a comprehensive indicator review. Additionally, many project activities directly refer to aligning with Sustainable Development Goals (SDGs), according to Interviewee 07 (I07). Core indicators, including Water footprint, Energy Carbon Footprint, Land Use Suitability Index (originally developed by FAO), and Climatic Risk indicators, were not only used but also expanded upon by the project, as highlighted by Interviewee 11 (I11). Interviewee 14 (I14) mentioned plans to create an indicator based on outcomes derived from the Decision Support System Tool. Lastly, Interviewee 15 (I15) noted the active development of a WEFE nexus indicator. Collectively, these insights underscore the multifaceted strategies and ongoing efforts to devise meaningful indicators that align with the complexities of the nexus assessment.

#### 3.1.7. Uncertainty or robustness analysis.

Exploring the subject of uncertainty and robustness analysis within the nexus projects considered in this study, a diverse array of approaches and insights, as drawn from the interviews, was revealed. Interviewees shared perspectives on the presence and application of uncertainty analysis. However, five of the projects did not contain any uncertainty analysis. A recurring observation is the need for Energy demand and supply to meet stringent requirements, often quantified at 99.9% reliability. For instance, Interviewee 02 (I02) employed Monte Carlo Analysis to address uncertainties in the complex interplay of energy dynamics. Interviewee 03 (I03) highlighted transparency as crucial, addressing uncertainty amidst numerous interdependencies through participatory processes. Similarly, Interviewee 05 (I05) utilized Probabilistic Projection Climate (PPC) scenarios, leveraging Monte Carlo Analysis to account for climate and societal uncertainties. Addressing climate scenarios, Interviewee 06 (I06) discussed the creation of scenarios based on climate models’ outputs, converting data into probability distributions using stochastic Monte Carlo sampling. This approach was complemented by sampling the range of distributions from AI and machine learning applications. Interviewee 08 (I08) adopted an ensemble of models to tackle uncertainty, enhancing the robustness of analysis for action areas. Interviewee 09 (I09) undertook resilience testing with a Monte Carlo analysis to evaluate circular solutions’ scalability and stress test their resilience under “black swan” disruptions. Robustness analysis found a place in Interviewee 10 (I10), employing Monte Carlo simulations. Interviewee 11 (I11) delved into both quantitative and qualitative analyses of water and energy dynamics, field data suitability, and system modeling. Uncertainty analysis extended to field data collection, as seen with Interviewee 12 (I12), who performed experimental campaigns with industrial processes. For Interviewee 13 (I13), the focus converged on climatic scenarios, encompassing uncertainty analysis.

#### 3.1.8. Challenges identified.

A series of challenges were mentioned by the interviewees, which can be categorized in 8 subgroups as follows:

***Awareness and understanding of the nexus***: Navigating the challenges surrounding the awareness and understanding of the nexus presents a multi-faceted attempt. Central to this is the need for a clear definition of the nexus itself, coupled with the task of identifying and quantifying the intricate trade-offs and synergies among its interconnected components. The absence of a universally recognized definition further compounds the challenge, as does the prevalent lack of comprehension among stakeholders regarding the nexus approach. Bridging this comprehension gap requires concerted efforts to foster an appreciation for the interconnections among the nexus components from both technical and cultural standpoints. Stakeholders’ demands for scientific evidence and clear recommendations pose a unique challenge, considering the nexus doesn’t offer a single optimal solution but rather serves as a tool to showcase diverse options within project capacities. Additionally, there’s a pressing need to generate awareness among policymakers, highlighting the significance of the nexus framework for informed decision-making. Altogether, addressing these challenges calls for a comprehensive approach that combines education, communication, and the recognition of the nexus as a versatile tool for sustainable development.

***Governance and decision-making process***: The subject of governance and decision-making within the nexus arena is fraught with several significant challenges. Major among these is the absence of policy alignment across the energy, water, and wastewater/effluent sectors, highlighting the need for cohesive strategies. Convincing policy stakeholders about the safety of repurposing treated water for agricultural use represents a specific hurdle that requires transparent communication. The lack of developed governance systems coupled with a limited understanding of the nexus’s opportunities and linkages further compounds the complexities. Transferring policy recommendations from theoretical realms to actionable decisions at higher echelons is a task that requires meticulous execution. National regulations often confine stakeholders within their boundaries, impeding fluid collaboration, and adapting to new bottom-up designed regulations proves to be a prolonged process. Additionally, shaping policy recommendations on a regional scale is challenging due to the divergent governance structures present across various nations within the action area. To surmount these challenges, a concerted effort is required to foster collaboration, develop effective governance systems, and encourage information sharing among stakeholders at multiple levels to enable well-informed, sustainable decisions within the nexus context.

***Interdisciplinary project work***: First and foremost, the imperative lies in establishing a cohesive connection and effective communication among diverse strands of nexus knowledge, encompassing domains like economics and biophysics. Achieving seamless communication of data and insights among the various disciplines engaged in a project emerges as a pivotal challenge. In this context, the creation of a shared foundation of knowledge or a comprehensive glossary proves indispensable for fostering a common understanding. The inclusion of stakeholders and their participation in specific project phases introduces a dynamic element but demands strategic management. Moreover, the task of harmonizing disparate languages, needs, focal points, and limited perspectives becomes paramount for comprehensive decision-making. Successfully addressing these challenges necessitates an orchestrated approach that hinges on effective communication strategies, fostering collaboration, and facilitating a holistic perspective that considers multiple dimensions of the nexus.

***Geographical application of projects***: The main challenge here is to emphasize on cross-border collaboration instead of limiting projects to national or regional levels.

***Complexity of connecting the nexus components***: The process of connecting the various components within the nexus presents a range of intricate challenges. Firstly, the system’s complexity demands the integration of numerous smaller models, necessitating a comprehensive approach. A significant hurdle lies in effectively transferring existing knowledge, information, and models from the realm of scientific theory to practical application. This includes the nuanced task of conveying insights from water modelers to water managers, for example. Moreover, certain aspects, such as biodiversity and ecosystem state, prove challenging to be modeled due to limited capacity. Incorporating ecosystem services into the models is another puzzle, especially when uniform valuation for these services is absent. The integration of outputs from various models and sectoral data introduces further complexity, demanding a harmonized approach. Organizing data for seamless usage across these models becomes crucial for successful integration. Challenges arise in integrated modelling, spanning scenario design, analysis, and their interconnectedness. Additionally, reconciling the energy sector with the water sector in the context of climate change adaptation presents a distinctive challenge. Lastly, bridging the gap between Water-Energy-Food-Ecosystems (WEFE) governmental authorities within countries becomes essential for effectively engaging policies into self-efficiency scenarios. In conclusion, the complexity of connecting nexus components requires innovative solutions to address these multifaceted challenges and promote holistic understanding and decision-making within the nexus framework.

***Data provision, availability, and sharing***: The very foundation of effective analysis hinges on factors such as data provision, accessibility, uncertainty, accuracy, reliability, and keeping data up-to-date. Furthermore, the integration of diverse data sources assumes critical importance. Ensuring the availability and accessibility of data and information stands as a pivotal concern, complicated by the fragmented institutional setup and disparate data management practices across the nexus sectors. The retrieval of specific data at the granularity of project scales, particularly at the river basin level, presents a unique challenge. Sharing data among stakeholders from various project components is essential for holistic analysis, yet it requires concerted efforts and strategic frameworks. A consistent theme is the paramount importance of obtaining proper data, which is further exacerbated by the necessity for data availability across the entire spectrum of the WEFE nexus nodes. Equally significant is the willingness of stakeholders to share data, which can influence the effectiveness of data-driven initiatives. Addressing these multifaceted challenges necessitates a cohesive approach involving collaboration, standardized data protocols, streamlined data collection methodologies, and a shared commitment to ensuring the accuracy and availability of data across the WEFE nexus landscape.

***Stakeholder engagement***: A significant hurdle lies in persuading industries to actively participate and showcase the benefits of technologies, a task that’s contingent upon the availability of data and measurements. A paradoxical situation emerges where industries need to participate to prove efficiency, yet their involvement hinges on demonstrated efficiency. Engaging industry stakeholders involves not only aligning them with project research goals but also addressing external issues affecting them, such as incidents like fires at treatment plants. Inclusivity extends to focus groups and Delphi studies, merging perspectives from local stakeholders and experts to form comprehensive insights. A pivotal concern revolves around demonstrating and adapting the financial viability of circular solutions, an undertaking that demands innovative strategies. Adding to the complexity, the challenge of acquiring data is closely linked to the need for initial funding, creating a cycle in which each becomes a prerequisite for the other. Navigating these challenges requires strategic negotiation, bridging the gap between industries and research, fostering inclusive dialogues, and pioneering ways to prove viability and secure resources, thereby promoting holistic stakeholder engagement within the WEFE nexus framework.

***Other***: Nexus projects introduce an array of challenges, including the dynamic aspect of project personnel changes and the subsequent shifts in focus driven by alterations in expertise. This challenge gains significance as personnel transitions can inadvertently lead to a re-evaluation of project goals and areas of emphasis. The seamless progression of projects hinges on the stability and consistency of the team, making abrupt personnel changes a potential source of disruption. A shift in focus is particularly evident when new team members bring diverse expertise to the table, necessitating adjustments to project priorities to leverage their unique strengths. This phenomenon underscores the need for agile project management strategies that can accommodate personnel changes without compromising the project’s trajectory. A comprehensive approach that blends flexibility, effective communication, and adaptable project planning is imperative to mitigate the potential impact of personnel changes and ensure a continuous and focused journey in nexus concepts.

The identification of these challenges, by linking strategies to specific negative or positive elements, led us to conduct a qualitative analysis of the interviews and specifically to the open-ended questions’ responses. This approach allowed us to explore in-depth the participants’ perspectives and experiences, uncovering the underlying elements behind the challenges and providing richer insights into the priorities for transcending disciplinary silos.

### 3.2. *Qualitative analysis results*

Based on the experiences shared by the interviewees, several aspects were discussed as either opportunities or challenges. Opportunities mainly refer to aspects viewed positively, either as achievements or anticipated accomplishments within the project. On the contrary, challenges refer to obstacles or negative aspects hindering the project’s progress.

In this context, Section 3.2 builds on and complements the quantitative synthesis of challenges presented in Section 3.1.8. While Section 3.1.8 provides an overview of the frequency and distribution of challenges across projects based on quantitative analysis, the qualitative analysis presented here explores how practitioners interpret and explain these challenges, the contexts in which they emerge, and their implications for WEFE implementation. Together, the two sections combine cross-project patterns with in-depth insights into practitioners’ experiences.

The following subsections elaborate on both opportunities and challenges, integrating interviewee responses where relevant.

**Fig 10:**
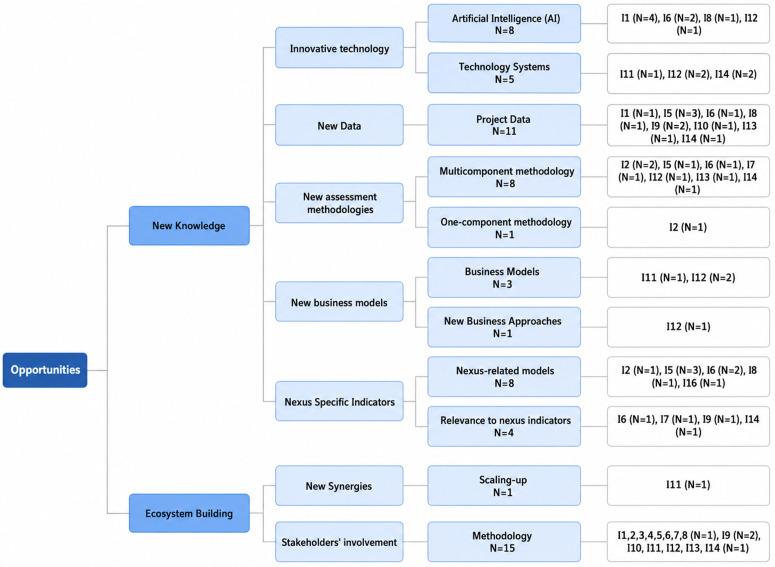
Opportunities dimension, themes, and sub-themes frequency based on the interviewees’ responses.

***Opportunities***: The theme of opportunities was predominant throughout the interviews, with 65 key mentions (Fig 10).

Many respondents highlighted the significant role of innovative technologies, especially artificial intelligence (AI) and machine learning, in improving sustainability and ecosystem management. Interviewees such as I1, I6, I8 and I12 emphasized AI’s ability to process large datasets, generate insights, and optimize solutions for sustainability challenges. AI was identified as a powerful tool for improving ecosystem management and predicting future scenarios, with respondents like I1 and I6 acknowledging that high-quality data is essential for training AI models effectively.

The development of new technology systems, especially those enhancing industrial resilience to climate change, was another area of opportunity identified by interviewees (I11, I12 and I14). Creating energy-efficient systems, such as water treatment technologies, was seen as crucial for reducing environmental impact and promoting sustainability. Circular systems, which integrate symbiotic processes across industries, were also identified as key opportunities to mitigate climate change effects and improve resource efficiency.

Data and its integration emerged as a central theme in the discussions of opportunities. Respondents (I5, I6, I8 and I11) discussed the value of using project data—particularly multi-model ensemble data and maps—when implementing sustainable practices. There was a shared consensus that data-driven decision-making was vital for managing climate change impacts effectively. Moreover, new assessment methodologies, such as multi-component approaches to evaluate Nexus interactions were considered valuable tools for measuring sustainability performance. Advanced techniques, which manage uncertainties in environmental data, were highlighted by interviewees (I5, I6) as key methods for improving prediction accuracy and informing sustainability strategies.

Further to this, interviewees (e.g., I11, I12) identified opportunities to create new business models fostering collaboration across sectors. One challenge, however, was aligning business models within the Nexus framework, given that each sector may have different objectives. The integration of circular economy principles into business models was seen as a promising approach to address both environmental and economic challenges, facilitating a more sustainable value chain.

Stakeholder involvement was considered another critical element for the project’s success. Interviewees (I2, I5, I8, I12) underscored the importance of engaging stakeholders, from policymakers to industry representatives, in the development and implementation of sustainable solutions. Methodologies for involving stakeholders varied, with respondents mentioning tools such as surveys, questionnaires, participatory workshops, and focus groups (I5, I6, I8, I11). These engagement strategies were viewed as essential for fostering collaboration, ensuring stakeholders’ diverse perspectives were considered, and achieving more inclusive decision-making.

Several respondents (e.g., I5, I2) also highlighted the use of institutional analysis to understand the roles and relationships of various stakeholders in sustainability efforts. Participatory workshops and living labs, where new sustainability solutions are tested in real-world settings, were noted as valuable for promoting community participation. Moreover, continuous stakeholder engagement throughout the project was emphasized (I6, I9), as it ensured that stakeholder feedback could refine strategies and align them with the needs and expectations of all involved parties.

**Fig 11:**
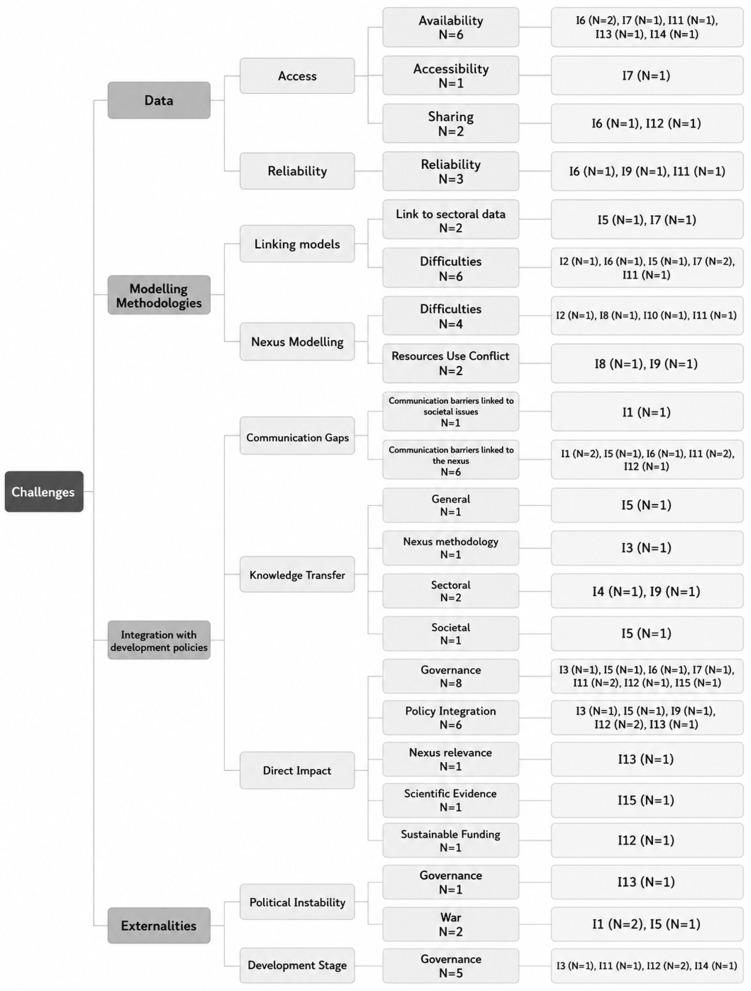
Challenges dimension, themes, and sub-themes frequency based on the interviewees’ responses.

***Challenges***: The challenges interviewees mentioned were related to multiple areas, such as data availability, modelling methodologies, stakeholder involvement, and governance systems (Fig 11). A total of 63 references were made concerning various challenges. A significant challenge that arose was the availability of data. Interviewees (e.g., I6, I13, I14) cited the difficulty of obtaining reliable and comprehensive data, particularly in countries with inadequate or non-existent public data collection systems. As I13 noted, despite these obstacles, the project moved forward due to the involvement of industries capable of providing necessary data. However, this often meant that the project was limited by the data available at any given time.

Data accessibility was another challenge, as highlighted by I7. Even when data was available, it was not always accessible to all relevant stakeholders, hindering the integration of data across project components. I6 and I12 raised concerns about data sharing, particularly with multinational corporations. These larger organizations were often reluctant to share data, whereas smaller companies were more inclined to collaborate. I12 observed that this reluctance from larger corporations significantly hindered effective collaboration and data integration.

Data reliability, particularly for long-term climate data, was another major issue. Interviewees (I6, I9, I11) noted the challenges of ensuring the accuracy of data spanning 50–100 years. I9 mentioned that climate projections over such long timeframes were difficult to validate, leading to discrepancies when comparing results across different projects. I11 further noted that while long-term climate data was valuable, validating it at local and regional scales was often impossible, adding further uncertainties to the project.

Aligning modelling methodologies with the project’s needs was another challenge discussed by several interviewees (e.g., I5, I7). While technology and data were rapidly advancing, applying these advancements to the WEFE Nexus framework was not straightforward. I5 explained that the challenge was not the technology itself but the difficulty of simplifying and transferring it into usable models for Nexus projects. The complexity of linking models to sectoral data made this even more challenging, as the models often failed to capture the system’s full complexity.

Interviewees (I2, I5, I6) discussed the difficulty of linking economic models with biophysical models. I5 explained that it was not just about publishing scientific models but making them practical for real-world use. The goal was not just to produce data but to provide actionable information for farmers and stakeholders on the ground. This required a level of integration that was difficult to achieve, especially when trying to bridge the gap between scientific modelling and practical application. I2 also emphasized that multiple models were required to capture the complexity of systems, making it difficult to create a cohesive and widely applicable model.

Specific challenges related to Nexus modelling were discussed by I2, I8, I10, and I11. I8 noted the difficulty of quantifying interdependencies between water, energy, food, and ecosystems. I2 raised concerns about the limited capacity to model aspects like biodiversity and ecosystem states, which are crucial to the Nexus framework but hard to model accurately. I11 added that aligning business models across different Nexus components was challenging, especially when sectors had competing goals and priorities.

Stakeholder involvement was another area of concern. I7 noted that engaging stakeholders at various stages of the project was difficult, especially in certain phases. Interviewees (I6, I7, I12) also pointed out deficiencies in governance systems, which hindered the progress of the project. In some countries, governance structures were underdeveloped, leading to ineffective collaboration among stakeholders. I12 elaborated that a lack of coordination between government ministries—such as the Ministries of Agriculture, Energy, and Water—complicated efforts to integrate policies across sectors.

Finally, governance and political stability were crucial to the project’s success. I3 emphasized that political stability played a significant role in ensuring the sustainability of project actions. In regions facing political instability, governance challenges hindered the effective implementation of policies and projects.

### 3.3. *Operational implications and pathways for improving WEFE implementation*

Building on the opportunities and challenges identified through the qualitative analysis, this subsection translates the empirical findings into a set of actionable pathways to support more effective implementation of the WEFE nexus in research and innovation projects. These pathways reflect practitioners’ perspectives and highlight areas where targeted interventions could enhance the operationalization of WEFE approaches.

First, strengthening conceptual clarity emerged as a key priority. Developing shared definitions, practical guidelines, and illustrative examples could support a more consistent understanding of WEFE principles across projects, sectors, and governance levels.

Second, improving cross-sector governance and policy alignment was identified as essential for addressing institutional fragmentation. At national and regional levels, this could involve the establishment of interministerial coordination mechanisms, shared policy roadmaps, and multi-level governance platforms that facilitate cooperation among water, energy, food, and environmental authorities.

Third, enhancing data accessibility and interoperability remains critical for robust WEFE analysis and decision-making. Investments in open data infrastructures, standardized data formats, and shared repositories would support data harmonization and improve the quality and transparency of WEFE modelling efforts.

Overall, these operational pathways illustrate how practitioner-identified challenges can be translated into constructive insights, thereby supporting the advancement of WEFE research and practice.

## 4. Conclusions

This study provides significant insights into the perspectives of project coordinators actively engaged in the water-energy-food-ecosystems (WEFE) nexus framework. Through structured interviews, we have gathered valuable empirical data that not only highlights the complexities of the nexus approach but also articulates the multifaceted benefits and challenges encountered in its implementation.

The interviews revealed several critical nexus components that are central to project coordinators’ work, notably water, energy, food, climate, and ecosystems. Respondents consistently emphasized the importance of recognizing the intricate interdependencies among these components to enhance resource efficiency and foster sustainability. A notable finding indicates that many projects are leveraging innovative technologies and nexus-specific indicators to better understand and manage these interconnections, reflecting a growing recognition of the necessity for advanced methodologies in this field.

Moreover, our analysis underscores the critical role of stakeholder engagement within the nexus framework. Interviewees highlighted how collaborative interactions with diverse stakeholders can lead to more effective project outcomes and decision-making processes. The emphasis on ecosystem building and inclusive practices points to an emergent trend of fostering synergies that extend beyond individual projects, thereby enhancing collective resilience against climatic and environmental challenges.

However, the study also illuminated significant barriers that hinder the full realization of the WEFE nexus potential. Challenges such as data availability and sharing, alignment of modeling methodologies, and the necessity of integrating project activities with broader development policies were identified as pressing issues. Interviewees articulated a clear need for improved governance frameworks that facilitate data access and sharing, allowing for comprehensive analyses and informed decision-making.

Considering these findings, it becomes imperative for policymakers, researchers, and practitioners to acknowledge and act upon the interconnected nature of water, energy, food, and ecosystems. Embracing an integrated approach to resource management not only enhances understanding but also leads to more effective policy interventions that align with sustainable development goals.

Ultimately, the insights garnered from our interviews provide a robust foundation for advancing the discourse on the WEFE nexus management. As this research highlights, addressing the complexities and challenges inherent in the nexus requires strategic collaboration, ongoing innovation, and a strong commitment to transparency and data sharing. By prioritizing these factors, stakeholders can work towards a resilient and sustainable future that harnesses the full potential of the WEFE nexus framework in tackling global resource challenges.

## Supporting information

S1 Appendix Structured interview questionnaire. The file contains the full interview protocol used in the study, including question IDs, question types, and possible response options.
(DOCX)
