# Supplementary material for: Advancing the WEFE nexus: Expert insights on implementation and challenges
Source: PLoS One. 2026 Jul 6;21(7):e0350133. doi: 10.1371/journal.pone.0350133 (PMC13336152; doi:10.1371/journal.pone.0350133)
Supplement: S1 Appendix — The file contains the full interview protocol used in the study, including question IDs, question types, and possible response options. (DOCX) [file pone.0350133.s001.docx]

**The structured questionnaire**

| Question ID | Question | Question type | Possible answers where applicable |
| --- | --- | --- | --- |
| Q1 | What are the nexus components your project deals with? | Multiple-choice | Water; Energy; Food; Ecosystem; Climate; Land & Soil; Waste; Health; Other |
| Q1.1 | Have you identified any nexus hotspots? (e.g., critical synergies and/or trade-offs) | Open-ended | n/a |
| Q2 | Aims for using the nexus approach | Multiple-choice | Environment and ecosystem; Human and resource security; Adaptation and resilience to climate change; Economic efficiency; Sustainability; Policy integration; Resource efficiency; Other |
| Q3.1 | Methodologies to model the nexus (Systems analysis) | Multiple-choice | System Dynamic Modelling SDM; Multi-sectoral system analysis; Material flow analysis; System informatics and analytics; Causal loop diagrams and system feedbacks; Mathematical/engineering modelling; Resource flows; Network analysis; Other |
| Q3.2 | Methodologies to model the nexus (Integrated modelling) | Multiple-choice | SWAT (Soil and Water Assessment Tool); CLEWS model (Climate, Land-use, Energy and Water Strategies); SEWEM (System-Wide Economic Water-Energy Model); WEF Nexus tool 2.0; PRIMA (Platform for Regional Integrated Modelling and Analysis); MCDA (Multicriteria Decision Analysis); MuSIASEM (Multiscale Integrated Analysis of Societal and Ecosystem Metabolism); Integrated assessment models; No relevant nexus method; Other |
| Q3.3 | Methodologies to model the nexus (Environmental management) | Multiple-choice | Scenario analysis; Footprinting; Life cycle assessment; Other |
| Q3.4 | Methodologies to model the nexus (Statistics) | Multiple-choice | Principal component analysis; Regression statistics; Trend analysis; Data mining; Other |
| Q3.5 | Methodologies to model the nexus (Economics) | Multiple-choice | Input-output analysis; Cost-benefit analysis; Trade-off/Synergy analysis; Social accounting matrix; Economic modelling; Value chain analysis; Supply chain analysis; Other |
| Q4 | Please name any models used in each of the nexus components that the project deals with | Open-ended | n/a |
| Q5.1 | Data used in quantifying the nexus | Multiple-choice | Sensors; Literature; Model output; Qualitative; Publicly available data platforms; National statistics; Other |
| Q5.2 | Publicly available data | Multiple-choice | EUROSTAT; FAO; OECD; COPERNICUS; Other |
| Q6 | Does your project include stakeholder engagement? | Multiple-choice | Institutional analysis; Questionnaires, surveys, or interviews; Historical analysis; Agent based modelling; Delphi technique; Critical discourse analysis; Stakeholder analysis; Participatory workshops/Focus groups; Living labs; Policy analysis; Other |
| Q7 | Please name any indicator developed or used in assessing the nexus | Open-ended | n/a |
| Q8 | Have you conducted uncertainty or robustness analysis? | Open-ended | n/a |
| Q9 | What are the challenges you identified? | Open-ended | n/a |
| Q10 | Additional questions & comments | Open-ended | n/a |
